# Computer-Based Executive Function Training for Combat Veterans With PTSD: A Pilot Clinical Trial Assessing Feasibility and Predictors of Dropout

**DOI:** 10.3389/fpsyt.2019.00062

**Published:** 2019-03-01

**Authors:** Ashley N. Clausen, Joan Thelen, Alex J. Francisco, Jared Bruce, Laura Martin, Joan McDowd, Robin L. Aupperle

**Affiliations:** ^1^VA Mid-Atlantic MIRECC, Durham VA Medical Center, Durham VA, Durham, NC, United States; ^2^Duke University Medical Center, Brain Imaging and Analysis Center, Duke University, Durham, NC, United States; ^3^Laureate Institute for Brain Research, Tulsa, OK, United States; ^4^Department of Psychology, University of Missouri-Kansas City, Kansas City, MO, United States; ^5^Department of Preventative Medicine and Public Health, University of Kansas Medical Center, Kansas City, KS, United States; ^6^Department of Community Medicine, University of Tulsa, Tulsa, OK, United States

**Keywords:** cognitive training, posttraumatic stress, executive function, fMRI, neuropsychological, cognitive inhibition, trauma treatment, placebo-controlled trial

## Abstract

**Background:** While evidence-based PTSD treatments are often efficacious, 20–50% of individuals continue to experience significant symptoms following treatment. Further, these treatments do not directly target associated neuropsychological deficits. Here, we describe the methods and feasibility for computer-based executive function training (EFT), a potential alternative or adjunctive PTSD treatment.

**Methods:** Male combat veterans with full or partial PTSD (*n* = 20) and combat-exposed controls (used for normative comparison; *n* = 20) completed clinical, neuropsychological and functional neuroimaging assessments. Those with PTSD were assigned to EFT (*n* = 13) or placebo training (word games; *n* = 7) at home for 6 weeks, followed by repeat assessment. Baseline predictors of treatment completion were explored using logistic regressions. Individual feedback and changes in clinical symptoms, neuropsychological function, and neural activation patterns are described.

**Results:** Dropout rates for EFT and placebo training were 38.5 and 57.1%, respectively. Baseline clinical severity and brain activation (i.e., prefrontal-insula-amygdala networks) during an emotional anticipation task were predictive of treatment completion. Decreases in clinical symptoms were observed following treatment in both groups. EFT participants improved on training tasks but not on traditional neuropsychological assessments. All training completers indicated liking EFT, and indicated they would engage in EFT (alone or as adjunctive treatment) if offered.

**Conclusion:** Results provide an initial framework to explore the feasibility of placebo-controlled, computerized, home-based executive function training (EFT) on psychological and neuropsychological function and brain activation in combat veterans with PTSD. Clinical severity and neural reactivity to emotional stimuli may indicate which veterans will complete home-based computerized interventions. While EFT may serve as a potential alternative or adjunctive PTSD treatment, further research is warranted to address compliance and determine whether EFT may benefit functioning above and beyond placebo interventions.

## Introduction

Post-traumatic stress disorder (PTSD) is a debilitating trauma-related disorder encompassing psychological and cognitive complaints (1, 2). PTSD is highly comorbid with other psychological problems including depression, substance use (2), and suicidal ideation (3). After the onset of Operation Iraqi Freedom (OIF) in 2003, the prevalence of combat-related PTSD rose dramatically from 2–3 to 23% (4, 5), highlighting the need to identify effective PTSD treatments.

Currently, the Veteran's Health Administration is disseminating two evidence-based psychological treatments for PTSD, including Prolonged Exposure (6) and Cognitive Processing Therapy (7). Despite empirical support, only 23–40% of veterans seek out psychological treatment (8). Of those who do engage in treatment, up to 60% continue to experience clinically significant symptoms (9). Further, evidence-based PTSD treatments do not directly target related cognitive and neuropsychological deficits including alterations in processing speed, executive functions, and verbal memory (1, 10).

Executive functions, including cognitive inhibition and attention regulation, are thought to be particularly important for emotional regulation in the context of PTSD (1, 11). Observed biases toward trauma-relevant stimuli and the hyperarousal symptoms of PTSD may partially stem from deficits in inhibition, disengagement and attentional control (1). Individuals with PTSD exhibit dysfunction in overlapping regions of the prefrontal cortex (PFC) during both emotional and executive function tasks, including the rostral anterior cingulate cortex (ACC) and dorsolateral PFC (1, 10). Propensity to recruit these regions relates to neuropsychological performance (12) and likelihood of treatment response (13). Therefore, treatments targeting executive functions and underlying neural processes may not only improve neuropsychological performance, but also beneficially impact psychological symptoms of PTSD.

Interference control training, training attention or working memory in the context of affective stimuli, is one such treatment that targets inhibitory processes, and has shown promise. In sexual assault survivors with PTSD, interference control training was associated with improved cognitive performance post-training, compared to those in a control training, and both trainings were associated with reduced PTSD symptoms (14). Similarly, in veteran populations, attentional control training (balance of attention toward threat and neutral stimuli) related to reductions in attention bias variability compared to attention bias modification training (directing attention away from threat); however, both trainings were associated with PTSD symptom reduction (15). While it holds promise as a PTSD intervention, it is unknown if cognitive training (e.g., interference control or attentional control training) is associated with improvements in overall executive or neuropsychological functioning or if broader-based executive control training would have similar or additional beneficial effects.

Another strategy is to target a range of executive functions and underlying neural processes using computer-based executive function training (EFT). Growing literature supports the potential utility of EFT with clinical populations. In depression, computer-based EFT combined with social skills training and group activities has been associated with improvements in memory encoding and retention compared to waitlist (16, 17). Computer-based EFT targeting visual, auditory, and cross-modality tasks was associated with decreases in depressive symptoms and increases in global executive control and attention (18). Additionally, training in selective attention and working memory led to greater decreases in depressive symptoms, rumination, decreased amygdala reactivity during emotional processing and increased dlPFC activity during working memory compared to treatment as usual (19). While EFT has shown some promise, placebo-controlled trials have not yet been conducted. Further, the feasibility and benefit for individuals with PTSD are less understood.

The present pilot study utilized a placebo-controlled design to determine the feasibility and acceptability of home-based, computerized EFT for combat veterans with PTSD. Given higher than expected dropout, we specifically examined predictors of treatment completion. In addition, we explored the potential effects of training on PTSD symptoms, neuropsychological performance, and neural activation during both emotional and cognitive processing paradigms. We expected EFT to be well-accepted and easily completed by veterans, and to result in improved clinical symptoms, neuropsychological function, and increased ACC and dorsolateral PFC activation during emotional and cognitive processing.

## Methods

### Participants

Participants were recruited from a community sample between August 2012 and June 2014 and included 52 male combat veterans who served since OIF. Participants were recruited via advertisements in the general community (i.e., radio, newspaper, and Facebook) and on local college campuses (i.e., via emails, flyers, etc.), and by providing informational flyers to clinicians at local VA hospitals. Veterans were excluded from the current study if they endorsed a psychological disorder other than PTSD as the primary cause for distress, current substance or alcohol use disorder, schizophrenia or bipolar disorder [determined via the Mini International Neuropsychiatric Inventory (20)], medical conditions affecting the hemodynamic response, current use of opioid's, benzodiazepines and/or thyroid medications, history of moderate to severe head injury (loss of consciousness >30 min, or post-traumatic amnesia lasting >24 h) or neurological disorder, or metal or devices contraindicated for fMRI. Veterans taking a stable dose (>6 weeks) of antidepressants or sleep medication were included. Two PTSD participants and one combat-exposed control participant reported taking a stable dose of antidepressants at baseline and denied changes in medication during treatment and at follow-up. Twenty veterans (*n* = 14 with PTSD) endorsed history of mild TBI.

**Figure 1:**
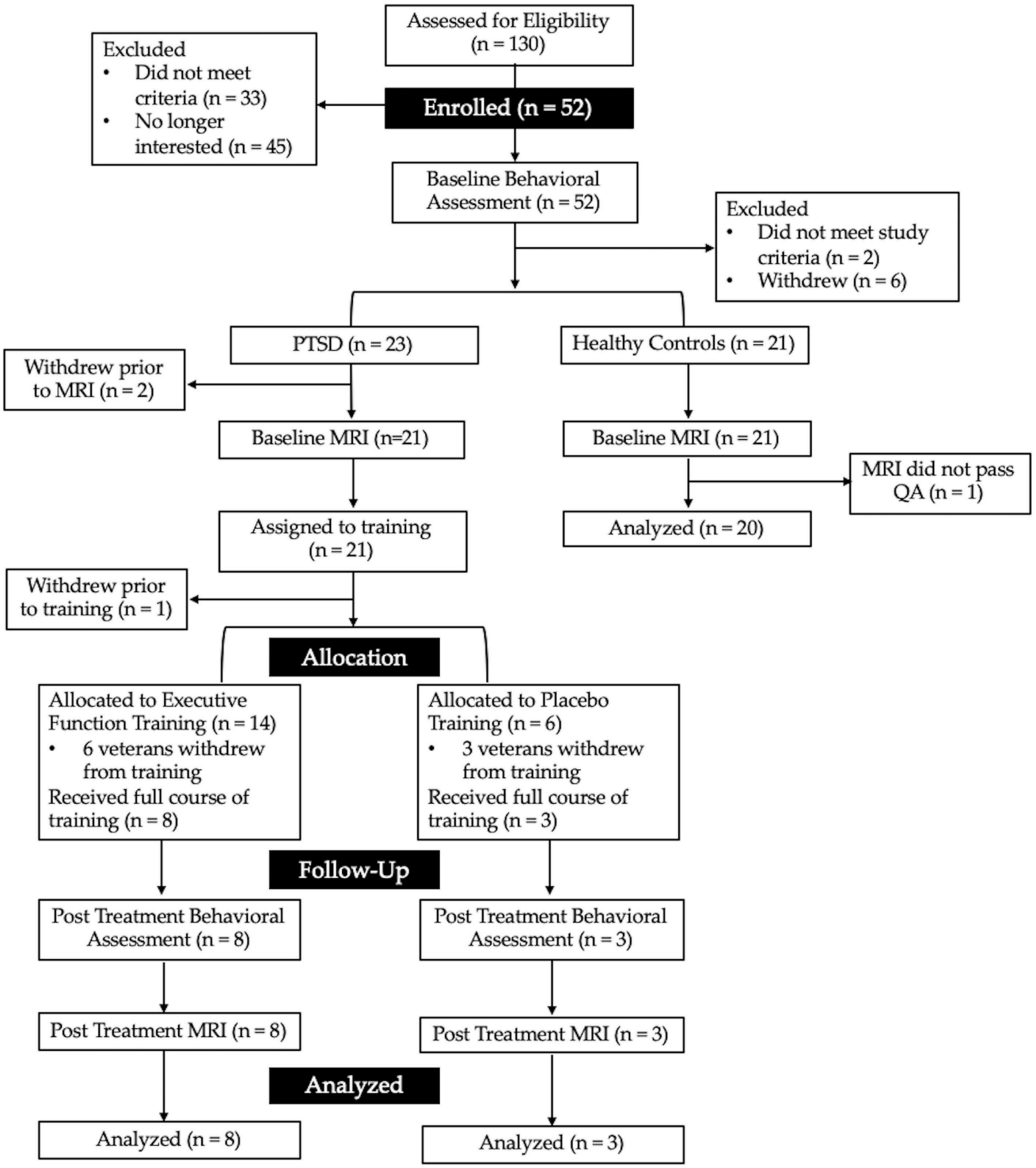
CONSORT diagram showing the flow of recruitment and retention for the present study. Interested veterans completed a phone screen to determine initial eligibility for the baseline behavioral assessment. Veterans were then assigned to either the combat-exposed control group (those without a current mental health disorder) or those with post-traumatic stress disorder (PTSD). Those with PTSD were assigned to 6-weeks of either executive function or active placebo training.

Following baseline assessment, two veterans did not meet study criteria and eight withdrew prior to completing the neuroanatomical assessment. Forty-two veterans, 21 with PTSD, and 21 without PTSD, completed the neuroanatomical assessment (described below). However, one MRI scan was excluded from the combat-exposed control group as it did not pass quality assurance (see Figure 1 for CONSORT diagram, and Table 1 for descriptive statistics). In addition, one veteran was excluded from analyses of the multisource interference task (MSIT) conducted during fMRI because of loss of behavioral data during that task due to equipment failure. A second veteran was excluded from neuroimaging analyses due to a lesion identified in radiologic review within a region of interest (ACC).

**Table 1:** Descriptive statistics at baseline.

	**EFT group (*n* = 14)**	**Placebo group (*n* = 6)**	**Combat-exposed controls (*n* = 20)**
	**Mean (SD)**	**Mean (SD)**	**Mean (SD)**
Age	32.6 (6.9)	36.7 (6.8)	30.7 (7.6)
Years of education	14.1 (2.0)	13.2 (0.8)	14.8 (1.9)
Tobacco use (% using tobacco)	46.4%	83.33%	70.0%
CAPS total score	55.0 (21.7)	61.3 (20.3)	13.4 (9.2)
PTSD symptom checklist total score	46.4 (13.0)	48.7 (15.6)	25.5 (6.9)
Depressive symptoms	16.9 (10.3)	14.3 (6.1)	6.8 (6.3)
Neurobehavioral symptoms	26.8 (13.0)	22.2 (10.3)	10.6 (7.5)
Impulsivity	20.1 (6.0)	22.2 (7.7)	20.8 (5.0)
Executive functioning	0.1 (0.6)	0.3 (0.2)	0.2 (0.4)
NP functioning	0.1 (0.4)	0.3 (0.3)	0.2 (0.4)
**PSYCHOLOGICAL DIAGNOSES (% ENDORSED)**			
Major depressive disorder, lifetime	50.0%	50.0%	30.0%
Obsessive compulsive disorder	7.1%	0%	0.0%
Agoraphobia	50.0%	16.7%	0.5%
PTSD (full criteria)	85.7%	53.3%	0.0%
Generalized anxiety disorder	0%	0%	20.0%
Antisocial personality disorder	7.1%	0%	0.0%

*EFT, executive functioning training; CAPS, clinician administered PTSD scale-IV; NP, neuropsychological; Tobacco use included current use of smokeless tobacco and cigarettes*.

This study was approved by the University of Missouri–Kansas City and University of Kansas Medical Center Institutional Review Boards. All participants provided written informed consent. The present study is registered with ClinicalTrials.gov (NCT01644851). Baseline data from overlapping samples has been previously published (21–23).

### Psychological Assessment

All veterans completed baseline assessments. Veterans who completed EFT and placebo training were also assessed post-treatment. The Mini International Neuropsychiatric Inventory for DSM-IV (20) was administered to assess Axis-I disorders. Current PTSD diagnosis and severity were assessed using the Clinician Administered PTSD Scale (CAPS)—IV (24). Twelve veterans met full criteria and four met partial criteria for PTSD, which was determined as missing one symptom from clusters C or D (see Supplementary Material for scoring criteria). Veterans also completed the PTSD symptom checklist (PCL)—Military version (25), Beck Depression Inventory (BDI)—II (26), Neurobehavioral Symptom Inventory [NSI (27)], and Sensation Seeking Scale [SSS (28)]. All measures showed adequate to excellent internal consistency in the present sample (α_CAPS_ = 0.92, α_PCL_ = 0.95, α_BDI_ = 0.88, α_NSI_ = 0.89, α_SSS_ = 0.77). A 10-item post-treatment questionnaire was created to assess acceptability of the treatment protocol. This questionnaire included questions relating to the intervention delivery method, how interesting and beneficial they felt training was, and whether they would engage in the training if offered by clinics (see Supplementary Material for full wording of this questionnaire).

### Neuropsychological Assessment

Neuropsychological assessment included the Delis-Kaplan Executive Function System Color-Word Interference Test (inhibition and flexibility), Tower Test [planning, rule learning, and inhibition (29)], Symbol Digit Modalities Test [visual scanning, perceptual speed, motor speed, and memory (30)], Auditory Verbal Learning Test [verbal learning and memory (31)], Trail Making Test [visual scanning, sequencing, switching and motor speed (32, 33)], and Neuropsychological Assessment Battery Digits [verbal attention (34)]. Z-scores for each test were averaged to obtain an overall mean score for neuropsychological performance. A second composite score was created for executive function related performance. To limit the impact of practice effects on neuropsychological tasks, alternative forms were given, when available, for the post-treatment assessment (see Supplementary Material for full neuropsychological administration and scoring procedures). Those who completed training repeated neuropsychological assessment post-treatment.

### fMRI Procedures

Scanning was conducted on a Siemens 3.0 Tesla Skyra MRI scanner. A T1-weighted anatomical scan was acquired using a 3D MPRAGE sequence (TR/TE = 2,300/2 ms, flip angle = 8°, FOV = 256 mm, matrix = 256 × 256, 1 mm slices). To assess prefrontal activation in relationship to PTSD during emotional and cognitive tasks, veterans completed the Multisource Interference task [MSIT (35, 36)] and the emotional cued anticipation task (12, 37, 38) during the scan, which were conducted similarly to prior studies. Briefly, the MSIT was developed specifically to assess cognitive inhibition [fMRI (35)]. The MSIT involves presentation of three digits and participants are instructed to identify the target digit that differs from the rest, using a button box. In congruent trails, the target location matches button position (e.g., XX3); for incongruent conditions, the target location does not match button position, requiring inhibition of the response to the number location (e.g., 311). Veteran's also completed an emotional cued anticipation task (12, 37), which combines a continuous performance task (CPT) with the interspersed presentation of affective visual stimuli. Participants are instructed 1) to press a button corresponding to the direction of an arrow on the screen and 2) that when the background screen turns blue, accompanied by a 250-Hz tone, a positive image will soon appear (positive anticipation); whereas, when the background turns yellow, accompanied by a 1,000 Hz tone, a negative image will appear (negative anticipation). Anticipation periods last 6 s, image presentation lasts 2 s, and the baseline CPT task is interspersed for variable duration averaging 8 s. Total task duration is 580 s. Response accuracy and reaction times are obtained for the CPT during baseline and the anticipation periods.

Each task was conducted during one gradient echo BOLD scan (35/43 axial slices for MSIT/anticipation; TR/TE = 2,000/25 ms, flip angle = 90°, FOV = 220 mm, matrix = 80 × 80, slice = 3.5 mm, 5 skip; 326/290 volumes for MSIT/anticipation). EPI scans were aligned to anatomical scans, volume registered, and corrected for slice timing and motion. For both tasks, the multiple regression models included the following regressors of no interest: residual motion (roll, pitch, and yaw), white matter mask to control for physiological noise, and baseline and linear trends. Regressors of interest included congruent and incongruent trials for the MSIT task and negative (NA) and positive (PA) anticipation periods, and negative and positive image presentation (analyses focused on anticipation periods) for the anticipation task. For both tasks, percent signal change (PSC) was calculated by dividing the regressor of interest by the baseline, and data were spatially blurred, normalized to Talairach space, and resampled to 4 mm^3^. PSC was extracted for the following regions of interest (ROI's): bilateral insula, amygdala, and dorsal midfrontal cortex, as well as dorsal, ventral, and rostral aspects of the ACC (Figure S1). More details on fMRI ROI construction are included in the Supplementary Material.

### Computer-Based Interventions

Veterans with partial or full PTSD (*n* = 21) received EFT or placebo training. One veteran withdrew from the study prior to training onset. Therefore, a total of 20 veterans were allocated to treatment (see Figure 1 for CONSORT diagram). The first 13 veterans were randomly assigned by a lab manager using a block random allocation sequence in groups of three (stratified by comorbid depression and/or TBI), created by a co-investigator (JB). All other research staff, including those administering psychodiagnostic and neuropsychological assessments were blind to the sequence of allocation until the study ended. Given compliance issues in combination with early study termination (due to the PI, RLA, moving institutions), the last seven veterans were assigned to EFT to optimize collection of feasibility and acceptability data. Veterans in both conditions completed daily, in-home computerized training and weekly phone check-ins to assess clinical symptoms and troubleshoot training obstacles (e.g., motivation, technical difficulties).

#### Executive Function Training

EFT was delivered using Lumosity™ (lumosity.com), which offers training tasks specifically related to executive functions that are based on traditional neuropsychological measures, visually engaging, and increased difficulty based on performance. The combination of tasks included in the present study provided training in the following aspects of executive functioning: response inhibition, attentional and task switching, working memory, and processing speed. Veterans were given access to free Lumosity accounts for the purposes of this study. Similar to protocols in other populations (39, 40), training was completed ~30-min per day, five times per week, for six weeks, and involved 60 sessions, 560 games, and an estimated 900 training minutes. Tasks were completed in the same order for each veteran and included the following: Color Match, Lost in Migration, Brain Shift, Brain Shift Overdrive, Speed Match, Memory Match, Memory Match Overload, Penguin Pursuit, and Disillusion [see (41) and Supplementary Material].

#### Placebo Word-Game Training

Placebo training involved word searches, hangman puzzles, and crosswords administered on a website created for this study. This training provided a relatively well-matched placebo condition due to involving (1) the same frequency/duration of computer-based training as EFT, (2) cognitively active tasks, (3) a plausible intervention to veterans, but was (4) unrelated to the domains of function theorized to be important for PTSD.

### Statistical Analyses

Statistical analyses were completed in R Statistical Software Package (http://cran.r-project.org). Separate logistic regressions were used to examine baseline predictors of training completion and included (a) clinical measures (CAPS, BDI-II, NSI, and SSS; all variance inflation factors (VIFs) < 2.0), (b) age, education, and neuropsychological function (all VIFs < 1.2), (c) ROI PSC during the MSIT, and (d) ROI PSC during the emotional anticipation task. Bonferroni correction resulted in a critical *p* ≤ 0.012. To assess the unique variance of each variable within the models, a *p*-value of 0.050 was used.

Separate linear mixed models were used to assess changes in each of the nine Lumosity training task brain processing index (BPI; a proprietary algorithm created by Lumosity to index task performance). Time was entered as both a fixed and random effect, and participant as a random effect. Bonferroni correction resulted in corrected critical *p*-values of 0.006.

To assess the potential benefits of training on measures of clinical symptoms, neuropsychological function, and brain function, we calculated z-scores for each subject that competed either EFT or placebo training (relative to the control group) for pre- and post-treatment. A z-score allowed for comparisons across measures, as well as the ability to compare individual changes in each domain (>1 z-score interpreted as potentially clinically significant). To inform power analyses for future studies, we report *t*-tests exploring time effects (combining EFT and placebo groups).

## Results

### Feasibility of Training

Dropout rates for EFT (*n* = 14) and placebo (*n* = 6) training were 42.8 and 50.0%, respectively. Within the EFT group, six veterans withdrew from the study (Table 2). One veteran withdrew prior to receiving training instructions, another veteran moved out of the state; the remaining four were unable to be reached for follow-up. Within the placebo group, three participants withdrew. One veteran indicated a lack of time and another indicated lack of motivation, frustration with the games, and “personal issues.” One veteran was unable to be reached for follow-up. On average, veterans completed 6.15 weeks of training, and completed an average of 535 individual games, equating to ~858 training minutes (Table 2).

**Table 2:** Executive functioning training summary.

**Participant**	**Completed?**	**Number of games completed (% of required training completed)**	**Weeks completed (#)**
1	Yes	548 (97.9%)	6
2	No	56 (10%)	3
3	Yes	560 (100%)	6
4	Yes	512 (91.4%)	8
5	Yes	560 (100%)	6
8	Yes	560 (100%)	6
9	No	22 (3.9%)	1
10	Yes	563 (100%)	6
11	No	0 (0.0%)	0
12	Yes	531(94.8%)	6
13	Yes	563 (100%)	7
14	No	0 (0.0%)	0
15	Yes	414 (73.9%)	8
16	No	0 (0.0%)	0

### Accessibility and Acceptance of Treatment

Eight veterans completed the EFT post-intervention questionnaire. Seven veterans agreed or strongly agreed the number of sessions, and duration of sessions were appropriate. All agreed or strongly agreed (a) with the delivery method of the intervention (computer-based training completed at home), (b) that the instructions were easy to comprehend, and (c) that the intervention was easy to access. Five veterans indicated preference for completing computer-based training at home, while three indicated no preference.

Six veterans agreed or strongly agreed that EFT beneficially impacted PTSD symptoms. All veterans felt EFT beneficially impacted their cognitive functioning. Seven veterans agreed or strongly agreed that EFT was fun and interesting. One veteran strongly agreed the intervention was boring and tiresome. When asked hypothetically if EFT was found to be effective for PTSD and offered as a treatment, one veteran indicated they would engage in the current treatment offered alone, while seven indicated they would engage in EFT combined with psychotherapy.

### Predictors of Treatment Completion

**Figure 2:**
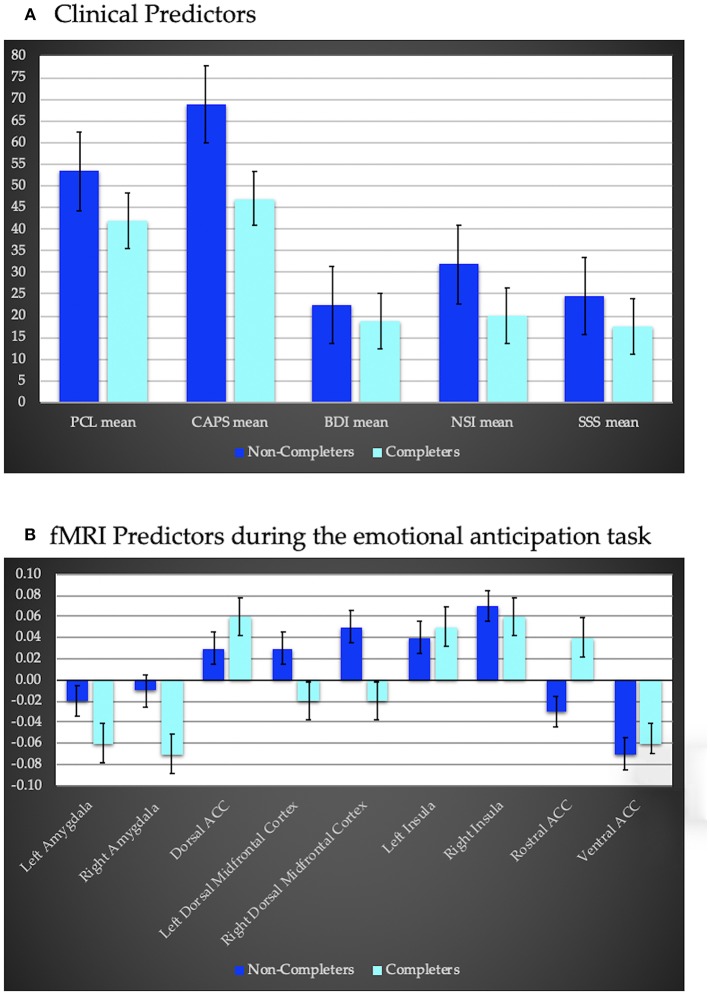
**(A)** clinical predictors of treatment completion including PTSD severity using the PTSD Symptom Checklist (PCL), and the Clinician Administered PTSD Scale (CAPS), depression severity assessed via the Beck Depression Inventory (BDI)—II, the Neurobehavioral Symptom Inventory (NSI) to assess sequelae of traumatic brain injury, and the Sensation Seeking Scale (SSS) to assess impulsivity. Completers exhibited less severe symptoms compared to non-completers. **(B)** fMRI predictors of treatment completion during an anticipation task (negative–positive affective trials). Regions of interest include bilateral amygdala, insula and dorsal midfrontal cortex, as well as dorsal, ventral, and rostral anterior cingulate cortex (ACC). Completers displayed hypoactivation within bilateral amygdala, and dorsal midfrontal cortex and hyperactivity within the rostral and dorsal ACC and left insula relative to non-completers.

Logistic regression indicated, when compared to the null model, the clinical predictors included in the model (PTSD, depressive, neurobehavioral and sensation seeking symptoms) yielded a better fit (AUROC = 0.94, LRT = −14.42, x^2^ = 18.47, *p* < 0.001; Figure 2A) where higher scores were positively associated with higher dropout rates. None of the clinical assessments were uniquely predictive of treatment completion. Cognitive variables were not predictive of treatment completion (AUROC = 0.67, LRT = −14.42, x^2^ = 2.64, *p* = 0.267).

PSC within ROI's during the anticipation task also significantly predicted treatment completion (AUROC = 1, LRT = −13.76, x^2^ = 27.53, *p* = 0.001; Figure 2B). Decreased activation within bilateral amygdala and bilateral dorsal middle frontal cortex, and increased activation within the dorsal, rostral and ventral ACC, and bilateral insula were associated with a higher probability of completion. None of the ROI's were uniquely predictive of treatment completion. PSC within ROIs during the MSIT did not predict treatment completion (AUROC = 0.88, LRT = −13.14, x^2^ = 10.08, *p* = 0.344).

### Pre- to Post-treatment Assessment

**Figure 3:**
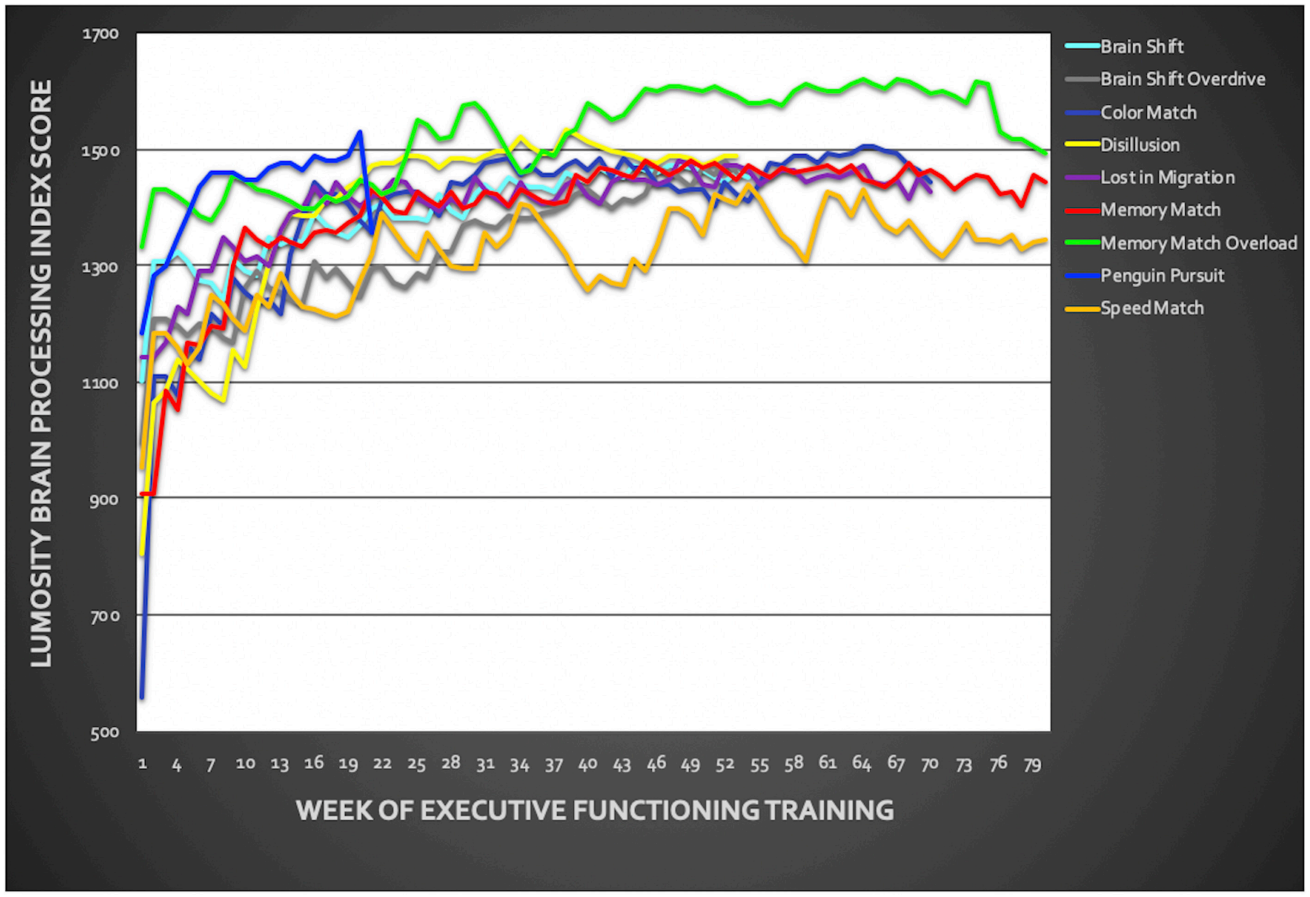
Training performance throughout the six-week executive function training. Training included the following Lumosity™ tasks: Color Match and Lost in Migration, Brain Shift, Brain Shift Overdrive, Speed Match, Memory Match, Memory Match Overload, Penguin Pursuit, and Disillusion. Overall, veterans showed improvement across all training tasks.

Veterans showed significant improvement in BPI across training tasks from pre- to post-EFT treatment (Figure 3; *p* < 0.001 for each task). Task results are presented in Figure 3 and Supplementary Material. On average, across both treatment groups, PTSD symptoms, measured via the PCL, improved by z = 1.28 with 7 out of 11 veterans demonstrating >1 z-score improvement from pre- to post-treatment. For depressive symptoms, an average improvement of *z* = 0.59 was observed, with only 3 out of 11 veterans demonstrating >1 z-score improvement from pre- to post-treatment. Veterans improved by *z* = 0.45 on neurobehavioral symptoms with 5 out of 11 veterans demonstrating >1 z-score improvement (Table 3). Changes in PSC within ROIs during the anticipation task and the MSIT are presented in Supplementary Materials (Table S1). However, no specific patterns of change emerged from pre- to post-treatment.

**Table 3:** Changes in psychological and neuropsychological scores from pre- to post-treatment.

	**Participant**	**CAPS**	**PCL**	**BDI-II**	**SSS**	**NSI**	**Executive function**	**NP function**
Placebo group (average)		−1.26	−0.34	−1.07	−0.73	−0.04	0.22	0.06
	4	−0.69	0.14	−0.8	−1.19	0.54	1.03	0.36
	6	−2.75	0.14	−0.64	−2.18	−1.34	−0.48	−0.24
	7	−0.34	−1.31	−1.76	1.19	0.67	0.1	0.06
EFT group (average)		−0.06	−1.25	−0.42	0.23	−0.6	−0.42	0.3
	1	0.57	−1.16	−0.32	0.59	1.07	−1.29	−0.21
	3	0.46	−2.33	−0.32	0.79	−0.94	0.76	0.75
	5	0	−1.89	−0.16	0.99	−1.07	0.03	−0.12
	8	−0.12	−1.31	−1.6	−0.4	−1.07	−0.69	0.48
	10	0.11	−0.44	0	−1.58	−0.94	−1.05	−0.78
	12	−5.73	−4.37	−1.44	NA	−2.54	−1.43	0.38
	13	1.95	−1.89	0.48	−0.59	−0.54	−1.07	0.61
	15	2.29	0.29	0	1.78	1.2	1.38	1.29

*EFT, executive function training; CAPS, clinician administered PTSD scale; PCL, PTSD symptom checklist-IV-military version; BDI-II, beck depression inventory II; SSS, sensation seeking scale; NSI, neurobehavioral symptom inventory; NP, neuropsychological. Negative values indicate a decrease in symptom severity from pre- to post-treatment*.

Specific to psychological symptomology, veterans showed a significant reduction from pre- to post-treatment in PTSD [*t*_(10)_ = 3.12, *p* = 0.011] and depressive [*t*_(10)_ = 2.71, *p* = 0.022] symptoms. Based on visual inspection and exploratory *t*-tests, changes in PTSD and depression symptoms did not appear to differ substantially based on treatment group (all *p*'s > 0.100). However, this analysis was notably limited due to the small sample sizes. There were no significance differences between pre- and post-treatment on measures of neurobehavioral symptoms, impulsivity, executive function, overall neuropsychological function, or PSC in any ROI (all *p*'s > 0.100).

## Discussion

The present pilot study examined the feasibility and acceptability of computer-based EFT for combat veterans with PTSD. Results provide several considerations that can inform future research. First, veterans who completed EFT indicated it was enjoyable and they would consider it as an adjunctive treatment for PTSD if offered. Second, EFT dropout rates were similar to traditional PTSD interventions (9). Third, clinical symptomology and brain activation during the anticipation task were predictive of treatment completion. Last, veterans who completed either EFT or placebo training showed improvements in clinical symptomology.

### Treatment Compliance

Prior studies assessing compliance for evidence-based psychotherapy PTSD treatments suggest ~39% dropout (42). However, the hope has been that non-trauma focused, computerized, at home treatments may lead to higher compliance (19). In support of this, studies examining cognitive training in depression and TBI report relatively low dropout rates of 0–31% (17–19). The current study is the first to explore computer-based training in combat veterans with PTSD. We found a 42.8% dropout rate for EFT suggesting a similar dropout rate to traditional PTSD treatments. Future research is warranted to explore modifiable factors that may influence EFT compliance in PTSD populations, such as having a designated time and place to engage in EFT, the dosing of training (frequency or duration of sessions), or treatments aimed at enhancing motivation (i.e., motivational interviewing, psychoeducation).

Prior research examining symptom severity as a predictor of completion for evidence-based PTSD psychotherapy has been mixed (42–44). Our results indicate that veterans with more severe symptoms are less likely to complete computerized training. The present sample included a relatively wide variability of symptom severity in a community-based veteran sample, and assessment encompassed a range of psychological symptoms—which may have enhanced our ability to detect this relationship. Given that none of the predictors contributed a unique amount of variance, findings suggest psychological severity across symptoms (e.g., PTSD, neurobehavioral) may be predictive of treatment completion.

Higher pre-treatment dorsal ACC, insula and amygdala activation during emotional anticipation has been reported to relate to PTSD treatment completion (45, 46). Our results highlight similar regions though differed in directionality. Specifically, results suggest that a balance toward recruitment of medial PFC regions involved in response inhibition or more implicit regulation, rather than the amygdala (affective or salience processing) or lateral PFC regions (executive functions, and *explicit* emotion regulation), may support the ability to stay committed to completing computerized cognitive interventions [for review of the function of these regions in PTSD see (47)]. Notably, average correlations between ROI activations and clinical measures were *r* = 0.24, suggesting activation patterns were not simply reflecting symptom severity. It would be beneficial if future research corroborated unique predictors for completion of computerized cognitive training vs. trauma-focused therapy, as this could point toward a personalized medicine approach.

### Acceptance and Impact of EFT

Overall, veterans who completed EFT felt the training modality was easily accessible, enjoyable, and liked that training was completed at home. Veterans subjectively reported that EFT beneficially impacted PTSD symptoms and cognitive abilities. While all veterans reported a desire to engage in EFT if offered in a clinical setting, most indicated a preference for EFT in conjunction with psychotherapy. Given these results and concerns about generalization of cognitive training effects, it may be beneficial to conduct EFT in conjunction with therapy discussions (i.e., concerning relevance of training for daily functioning or PTSD symptoms). Anecdotally, veterans demonstrated variability in their reflections about the impact of completing EFT. One veteran in particular noted connections between learning to slow down and inhibit automatic responses on the training tasks with learning to do the same in his daily life (e.g., stopping himself from yelling at a loved one and instead, responding in a more adaptive way). However, others would indicate how they liked the training but did not understand how it was related to their PTSD symptoms. Implementing cognitive training within a therapeutic context could potentially enhance the impact by making these connections between training tasks and every day functioning.

Veterans in both groups showed PTSD and depressive symptom reductions from pre- to post-treatment and the EFT group showed significant improvement on Lumosity trainings tasks. However, obvious benefits were not observed in neuropsychological function, self-reported neurobehavioral symptoms, or brain activation within this small sample. Thus, it is possible that PTSD and depressive symptom improvement may be reflective of a placebo effect, or indicate that focused cognitive exercises, regardless of content, are beneficial. Future research is needed, with sufficient sample sizes, to further assess changes in psychological symptoms, cognitive function and brain activation following EFT compared to an active-placebo control.

### Limitations

Given the focus on male combat veterans, the current study cannot address acceptability and feasibility of EFT with other PTSD populations (females; non-combat types of trauma). The present study focused on a six-week home-based training protocol, similar to protocols in other populations. While more obvious clinical benefits may have emerged with a longer training protocol, there is no data to suggest the optimal dose for computer-based EFT. Additionally, while home-based treatments may increase accessibility to treatment, it may introduce additional limitations to treatment effects, such as less structured behavioral activation of coming to a clinic, or increased distractibility while at home. Due to the small sample, the current study is unable to assess efficacy of EFT compared to placebo training. Thus, the current results focus on feasibility and factors related to treatment completion. However, results from this study can be used to inform future studies examining the effects of EFT. Specifically, further research with larger samples is needed to identify whether computer-based EFT may have significant clinical benefit and if so, the optimal dose (i.e., number and duration of training sessions) and necessary components (i.e., working memory, attentional switching, etc.) of training.

### Conclusions

Despite limitations, our results provide an initial framework to explore the impact of EFT on psychological symptoms, neuropsychological function and brain activation. Results suggest that home-based, computerized EFT may have similar issues with compliance as other evidence-based PTSD treatments. Lower symptom severity and a balance toward medial PFC cognitive control regions rather than affective processing regions or lateral PFC regions during emotional anticipation may support the ability to complete such self-driven interventions. Veterans who completed training reported a high level of acceptance for EFT and suggested they would choose to complete such training in conjunction with other treatments. Initial findings suggest that EFT may relate to subjective and clinically significant improvement in PTSD and depressive symptoms, but the active treatment mechanism remains unclear. Future research is warranted to examine whether computerized EFT may be useful for augmenting current, evidence-based PTSD treatments and identifying strategies for improving compliance and efficacy of such interventions.

## Data Availability

The de-identified datasets for this study are available upon request.

## Author Contributions

JB, LM, JM, and RA contributed conception and design of the study. AC, JT, AF, and RA contributed to the acquisition of data. AC and RA performed the statistical analysis. AC wrote the first draft of the manuscript. RA and JT wrote sections of the manuscript. All authors contributed to manuscript revision, read, and approved the submitted version.

### Conflict of Interest Statement

LM reported receiving funding from the American Cancer Society (Principal Investigator), the University of Kansas Research Investment Council (Co-Investigator), the University of Kansas Cancer Center Pilot Program (Co-Investigator), and the National Institutes of Health (R01 HD086001 [Co-Investigator]; K23 GM123320 [Collaborator]). RA reported serving as consultant for a Department of Defense Congressionally Directed Medical Research Program project (PT100018) and has received funding from the National Institute of Health (K23MH108707). JB reported that he is a paid consultant to the National Hockey League and provides unbranded talks for Novartis. JM, AC, AF, and JT reported no commercial or financial relationships that could be construed as a potential conflict of interest.
