# Supplementary material for: Computer-Based Executive Function Training for Combat Veterans With PTSD: A Pilot Clinical Trial Assessing Feasibility and Predictors of Dropout
Source: Front Psychiatry. 2019 Mar 1;10:62. doi: 10.3389/fpsyt.2019.00062 (PMC6405637; doi:10.3389/fpsyt.2019.00062)
Supplement: Supplementary file 1 [file Data_Sheet_1.docx]

Supplementary Material

Feasibility of Computer-based Executive Function Training for Combat Veterans with PTSD

**Ashley N. Clausen, Ph.D., Joan Thelen, M.A., Alex J. Francisco, M.A. , Jared Bruce, Ph.D., Laura Martin, Ph.D., Joan McDowd, Ph.D., and Robin L. Aupperle, Ph.D.**

*** Correspondence:** Dr. Ashley Clausen: Ashley.n.clausen@gmail.com

# Psychological and Neuropsychological Assessment Scoring

For PTSD diagnosis, based on the CAPS-IV, items that were scored as a two or higher on intensity and as a one or higher on frequency were counted as a symptom (Blake et al., 1995). Twelve veterans met full diagnostic criteria for PTSD (total severity score ≥ 30 and full criteria for each symptom cluster) and four met for partial PTSD (total severity score ≥ 30 but not meeting full criteria for symptom clusters C or D [missing 1 symptom]).

All subjects completed a battery of neuropsychological assessments that included the Delis-Kaplan Executive Function System Color-Word Interference Test (word reading, color naming, inhibition, and inhibition/switching completion times), Tower Test (Total Achievement Score; (Delis et al., 2004)), Symbol Digit Modalities Test (total correct; (Smith, 1982)), Auditory Verbal Learning Test (trials 1-5 total correct; (Strauss et al., 2006)), Trail Making Test (time to completion for Trails A and B (Reitan, 1979; Wagner et al., 2011), and Neuropsychological Assessment Battery Digits (forward and backward (Reitan and Wolfson, 1985)). Raw scores were transformed into scaled scores based on previously published norms for each assessment, respectively. These scaled scores were converted into z-scores and the z-scores were then averaged to obtain an overall mean score for neuropsychological performance. To assess the unique impact of executive functioning, a second composite score was created using Color-Word Interference Test scales of Inhibition (controlling for reading and naming of words) and Switching (controlling for inhibition), Symbol Digit Modalities Test, Trail Making Test B, and the Neuropsychological Assessment Battery Digits backwards. Those who completed training completed neuropsychological assessment again at post-treatment. To limit the impact of practice effects on neuropsychological tasks, alternative forms were given, when available, for the post-treatment assessment. Alternative forms were available for the Auditory Verbal Learning Test, Trail making Test, Neuropsychological Assessment Battery Digits, and the Symbol Digit Modalities Test.

# Description of Lumosity Training Games

Briefly, “Color Match” is based on the Stroop Task in which two cards are presented simultaneously. A word is presented on the left and subjects must identify if the meaning of the word on the left matches the color of the text presented on the right (ignoring the content of the word on the right). “Lost in Migration” is a Flanker task in which subjects use a button to indicate which direction the center bird is facing in a line of birds. “Brain Shift” is an attentional switching task in which subjects switch between identifying if a card number is odd or even and if a letter is a vowel or consonant depending on where the card appears on the screen and includes two stacks of cards. “Brain Shift Overdrive” is very similar but includes four stacks of cards versus two. “Speed Match”, “Memory Match”, and “Memory Match Overload” are similar to n-back tasks. In “Speed Match” subjects are asked to identity if a stimulus matches the stimulus presented immediately prior, assessing processing speed. In “Memory Match” and “Memory Match Overload”, subjects are asked to identify if the stimulus on the screen matches the one presented two (2-back) or three (3-back) stimuli previously, engaging working memory. “Penguin Pursuit” assess visuiospatial abilities and task switching by requiring the subject to navigate a penguin through a maze as the maze rotates its orientation (thus, the right arrow button will take the penguin different directions in the maze depending on the rotation). Lastly, “Disillusion” is an attention-shifting task in which subjects are required to place puzzle pieces based on either the color or shape of symbols appearing on the puzzle piece, depending on the orientation of the puzzle (i.e., wide versus tall).

# Post-Treatment Questionnaire

The Post-Treatment Questionnaire is a self-report measure that is comprised of 10 items and was created to assess acceptability of the treatment protocol. Eight of the items were rated on a Likert-scale assessing level of agreement. A “1” indicated, “very much disagree”; a “2” indicated, “disagree”; a “3” indicated, “neutral”; a “4” indicated, “agree”; a “5” indicated, “very much agree”. The following items were rated on the above scale: 1) “I felt the number of sessions was appropriate”; 2) “I felt the length of the sessions were appropriate”; 3) “I was satisfied with the method (i.e., web-based system) of accessing the intervention”; 4) “I understood instructions on how to access and complete intervention”; 5) “I felt this program was helpful in reducing my PTSD symptoms”; 6) “I felt this intervention was helpful for enhancing my cognitive skills”; 7) “The intervention was fun and interesting to complete”; 8) “The intervention was boring and tiresome to complete”. One item rated preference of intervention delivery and was phrased as follows: “I would have preferred the intervention be delivered…”. This item was rated on the following scale: a “1” indicated, “at home”; a “2” indicated, “in the investigators office”; a “3” indicated, “no preference”. The last item assessed the participants’ willingness to engage in EFT: “If this intervention was found effective for symptoms of PTSD and was offered as treatment at a clinic (i.e., without compensation), I would likely”. The following options were provided: a “1” indicated, “choose to complete this intervention”; a “2” indicated, “choose a talk or behavior therapy that had been shown effective”; a “3” indicated, “choose a medication treatment”; a “4” indicated, “choose both the current intervention and talk/behavior therapy”; a “5” indicated, “choose both the current intervention and medication”; a “6” indicated, “choose both medication and talk/behavior therapy”; a “7” indicated, “choose to complete all three (this intervention, medication, and talk/behavior therapy).”

# Construction of Optimized Anatomical Region of Interest Masks

Region of interest (ROI) masks were constructed using a data-driven combination of information from Talairach stereotactic definitions and anatomical gray matter probabilities in order to increase the likelihood that ROI boundaries mapped onto regional gray matter following mask dilation. In brief, 43 high-resolution T1-weighted images (172 sagitally acquired spoiled gradient recalled 1mm thick slices, inversion time (TI = 450 msec, TR = 8 msec, TE = 4 msec, flip angle = 12 degrees, FOV = 250 x 250 mm) were acquired from healthy adult participants without any existing or prior psycho/neuropathology. These anatomicals were parcellated into gray and white matter using the standard segmentation protocol from SPM5 (Statistical Parametric Mapping software; <http://www.fil.ion.ucl.ac.uk/spm>) implemented in Matlab 7.5.0 (MathWorks, Natick, Massachusetts). Individual gray matter maps were then normalized to Talairach stereotactic space and combined to produce a voxelwise gray matter probability map. Thus, each voxel contained the *a-priori* probability of assignment to gray matter as determined by the SPM5 segmentation protocol across the 43 healthy participants. This gray matter probability map was then combined with Talairach stereotactic definitions to yield 76 Talairach-defined brain regions with voxelwise gray matter probabilities. Different gray matter probability thresholds (ranging from 5-50% in increments of 5) and dialation (in downsampling from 8.0mm^3^ to 64.0mm^3^ voxels) clip level (ranging from 5-50% in increments of 5) maps were then produced for each Talairach region, and the overlap of each probability/clip level map with the stereotactic atlas definition was assessed through computing sensitivity and specificity calculations. These sensitivity/specificity values were then plotted on a receiver-operator curve, and the gray matter probability/clip level which individually maximized sensitivity/specificity for each particular Talairach region was chosen as the optimal probability/clip level for that region. Bilateral insula (Right = 19,264 mm^3^; Left = 19 712 mm^3^), amygdala (Right = 1728 mm^3^; Left = 1664 mm^3^), and middle frontal (Right = 42,944 mm^3^; Left = 43,584 mm^3^) regions were included in the 76 regions identified. The region for the anterior cingulate was separated The anterior cingulate regions were further separated into ventral (z≤0; 10,240 voxels for ventral ACC) and rostral (z>0; 11,456 voxels for rostral ACC) aspects based on Talairach coordinates. The dorsal ACC region was constructed based on the anterior portion of the dorsal cingulate (y<0; z>25; 13,760 voxels). The ROIs are displayed in Figure S1.

# Training Performance

Overall, EFT veterans showed improvement in BPI for all training tasks from beginning to end of training (Figure 3). Related to processing speed, subjects improved on Speed Match (AIC = 7854.22, F = 284.89, LRT = -3922.11, *p* < 0.001). On tasks of working memory, subjects improved on Memory Match (AIC = 7597.63, F = 487.08, LRT = -3793.82, *p* < 0.001), and Memory Match Overdrive (AIC = 7143.36, F = 435.14, LRT = -3566.68, *p* < 0.001). On tasks assessing inhibition, subjects improved on Color Match (AIC = 7438.44, F = 444.27, LRT = -3714.22, *p* < 0.001) and Lost in Migration (AIC = 6760.34, F = 98.38, LRT = -3375.17, *p* < 0.001). Similarly, subjects showed improvement on Brain Shift, an attentional switching task (AIC = 5351.86, F = 283.01, LRT = -2670.932, *p* < 0.001), Brain Shift Overdrive (AIC = 5106.09, F = 501.35, LRT = -2548.045, *p* < 0.001), and Disillusion (AIC = 5050.41, F = 746.751, LRT = -2520.20, *p* < 0.001). Lastly, on a test of visuospatial and switching ability, subjects showed significant improvement on the Penguin Pursuit task (AIC = 1909.08, F = 150.57, LRT = -949.54, *p* < 0.001).

# fMRI Analysis from Pre- to Post-Treatment

For incongruent – congruent trails during the MSIT, subjects showed .82 z-score increase in activation from pre- to post-treatment within the right amygdala with 5 out of 11 subjects demonstrated > 1 z-score change (See Table S1). Within the ventral ACC during the MSIT, subjects showed an average increase in activation from pre- to post-treatment by .63 z-scores with 3 out of 11 subjects demonstrated > 1 z-score change. During the anticipation task, subjects showed decreased activation on negative – positive anticipation trials within the right amygdala (.70 z-score change), left dorsal midfrontal cortex (.49 z-score change), right insula (.88 z-score change) and rostral ACC (.50 z-score change), as well as increased activation within the right dorsal midfrontal cortex (.52 z-score change).

# References

Blake, D.D., Weathers, F.W., Nagy, L.M., Kaloupek, D.G., Gusman, F.D., Charney, D.S., et al. (1995). The development of a Clinician-Administered PTSD Scale. *Journal of Traumatic Stress* 8(1)**,** 75-90.

Delis, D.C., Kramer, J.H., Kaplan, E., and Holdnack, J. (2004). Reliability and validity of the Delis-Kaplan Executive Function System: an update. *J Int Neuropsychol Soc* 10(2)**,** 301-303. doi: 10.1017/S1355617704102191.

Reitan, R.M. (1979). *Trail Making Test: TMT.* Testzentrale.

Reitan, R.M., and Wolfson, D. (1985). *The Halstead-Reitan neuropsychological test battery: Theory and clinical interpretation.* Reitan Neuropsychology.

Smith, A. (1982). *Symbol Digit Modalities Test.* Los Angeles: Western Psychological Services.

Strauss, E., Sherman, E.M., and Spreen, O. (2006). *A compendium of neuropsychological tests: Administration, norms, and commentary.* USA: Oxford University Press.

Wagner, S., Helmreich, I., Dahmen, N., Lieb, K., and Tadic, A. (2011). Reliability of three alternate forms of the trail making tests a and B. *Arch Clin Neuropsychol* 26(4)**,** 314-321. doi: 10.1093/arclin/acr024.

# Supplementary Figures

**
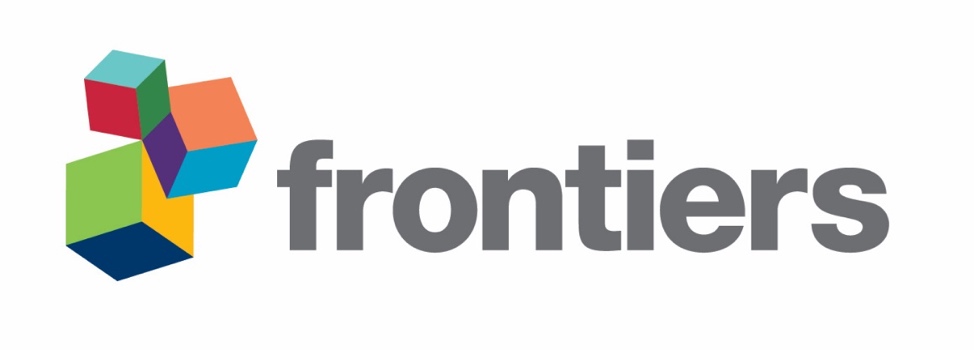
Supplementary Figure 1.** Anatomical regions of interest. Regions of interest include the ventral (yellow), rostral (yellow-orange) and dorsal (orange) aspects of the anterior cingulate, bilateral insula (z = 13), bilateral amygdala (z = -16), and bilateral middle frontal gyrus (z = 30).

Table S1. Changes in brain activation (percent signal change) during the MSIT and the anticipation task from pre- to post-treatment

|  | **ID** | **Left Amygdala** | **Right Amygdala** | **Dorsal ACC** | **Left Dorsal Midfrontal Cortex** | | **Right Dorsal Midfrontal Cortex** | | **Left Insula** | | **Right Insula** | | | **Rostral ACC** | | | **Ventral ACC** | |  |
| --- | --- | --- | --- | --- | --- | --- | --- | --- | --- | --- | --- | --- | --- | --- | --- | --- | --- | --- | --- |
| *MSIT Task (Incong-Cong Trials)* | | | | | | | |  | |  | | |  | | |  | | |  |
| Placebo Group (average) | | 0.17 | 1.26 | 0.12 | 0.42 | | 0.12 | | 0.47 | | | 0.34 | | | 1.27 | | | 1.96 | |
|  | 006 | -0.97 | 0.37 | -0.21 | 0.51 | | 0.76 | | -0.55 | | | -0.33 | | | 0.25 | | | -0.41 | |
|  | 009 | 1.26 | 2.25 | 0.79 | 0.94 | | -0.41 | | 1.03 | | | 0.00 | | | 2.21 | | | 5.11 | |
|  | 014 | 0.22 | 1.16 | -0.21 | -0.18 | | 0.01 | | 0.93 | | | 1.35 | | | 1.35 | | | 1.18 | |
| EFT Group (average) | | -0.34 | 0.64 | -0.44 | -0.22 | | -0.16 | | -0.05 | | 0.13 | | | -0.19 | | | 0.06 | |  |
|  | 002 | 0.21 | 1.32 | -0.29 | -0.32 | | -0.33 | | 0.88 | | 0.80 | | | 0.01 | | | -0.35 | |  |
|  | 005 | 0.08 | 0.18 | -0.19 | -0.01 | | -0.53 | | -0.45 | | 0.00 | | | -0.28 | | | -0.41 | |  |
|  | 008 | 0.03 | 0.61 | -2.24 | -1.94 | | -1.54 | | -1.48 | | -0.63 | | | -1.37 | | | 1.04 | |  |
|  | 015 | 0.10 | 1.24 | -0.28 | -0.51 | | 0.25 | | 0.53 | | 1.43 | | | -1.57 | | | 0.68 | |  |
|  | 018 | -0.38 | -0.34 | -0.12 | 0.62 | | 0.12 | | 0.28 | | -0.04 | | | -1.06 | | | -0.39 | |  |
|  | 024 | NA | NA | NA | NA | | NA | | NA | | NA | | | NA | | | NA | |  |
|  | 025 | -1.03 | 1.19 | -0.04 | 0.18 | | 0.23 | | 0.39 | | 0.62 | | | 1.14 | | | -0.53 | |  |
|  | 027 | -0.81 | 0.25 | 0.05 | 0.46 | | 0.69 | | -0.49 | | -1.30 | | | 1.79 | | | 0.39 | |  |
|  | | | | | |  | |  | |  | | |  | | |  | | |  |
| *Anticipation Task (NA-PA Trials)* | | | | | |  | |  | |  | | |  | | |  | | |  |
| Placebo Group (average) | | 1.29 | 1.34 | -2.84 | 0.67 | | -0.94 | | -0.04 | | 0.26 | | | 0.11 | | | -2.59 | |  |
|  | 006 | 2.26 | 2.08 | -3.40 | 0.92 | | -0.88 | | -0.19 | | -1.29 | | | 1.04 | | | 0.10 | |  |
|  | 009 | 0.26 | 1.43 | -3.16 | -0.43 | | -2.45 | | -0.87 | | 1.13 | | | -0.87 | | | -6.67 | |  |
|  | 014 | 1.35 | 0.52 | -1.96 | 1.53 | | 0.52 | | 0.94 | | 0.94 | | | 0.15 | | | -1.19 | |  |
| EFT Group (average) | | -0.34 | 0.46 | -2.18 | 0.42 | | -0.36 | | -0.13 | | 1.11 | | | 0.64 | | | 1.33 | |  |
|  | 002 | -3.47 | -0.71 | -1.25 | -0.67 | | -0.19 | | -0.92 | | 2.91 | | | 3.18 | | | 5.15 | |  |
|  | 005 | -1.03 | 1.42 | 0.08 | -0.34 | | 0.37 | | 1.29 | | 0.32 | | | -0.20 | | | 1.54 | |  |
|  | 008 | 0.27 | 1.17 | -2.76 | 1.44 | | 0.05 | | -1.40 | | 0.62 | | | 0.99 | | | 1.91 | |  |
|  | 015 | -0.91 | -0.49 | -3.23 | 1.23 | | -1.01 | | -2.27 | | 0.75 | | | 1.69 | | | 1.11 | |  |
|  | 018 | 0.36 | -0.53 | -0.80 | 0.08 | | -2.14 | | 2.08 | | 2.61 | | | 1.35 | | | 1.12 | |  |
|  | 024 | 0.70 | 0.03 | -5.21 | -0.72 | | 0.06 | | 0.18 | | -0.87 | | | -1.54 | | | 1.72 | |  |
|  | 025 | 0.99 | 1.35 | -2.08 | 3.00 | | 0.76 | | -0.31 | | 0.53 | | | 0.39 | | | -3.58 | |  |
|  | 027 | 0.39 | 1.43 | -2.19 | -0.63 | | -0.76 | | 0.31 | | 2.02 | | | -0.71 | | | 1.64 | |  |

**Note**: MSIT = Multi Source Interference Task; ACC = Anterior Cingulate Cortex; Incong-Cong = percent signal change during incongruent minus congruent trails; NA-PA = percent signal change during negative minus positive anticipation trials. Negative values indicate a decrease in activation from pre- to post-treatment.
