# Supplementary figures and images for: Computer-Based Executive Function Training for Combat Veterans With PTSD: A Pilot Clinical Trial Assessing Feasibility and Predictors of Dropout
Source: Front Psychiatry. 2019 Mar 1;10:62. doi: 10.3389/fpsyt.2019.00062 (PMC6405637; doi:10.3389/fpsyt.2019.00062)

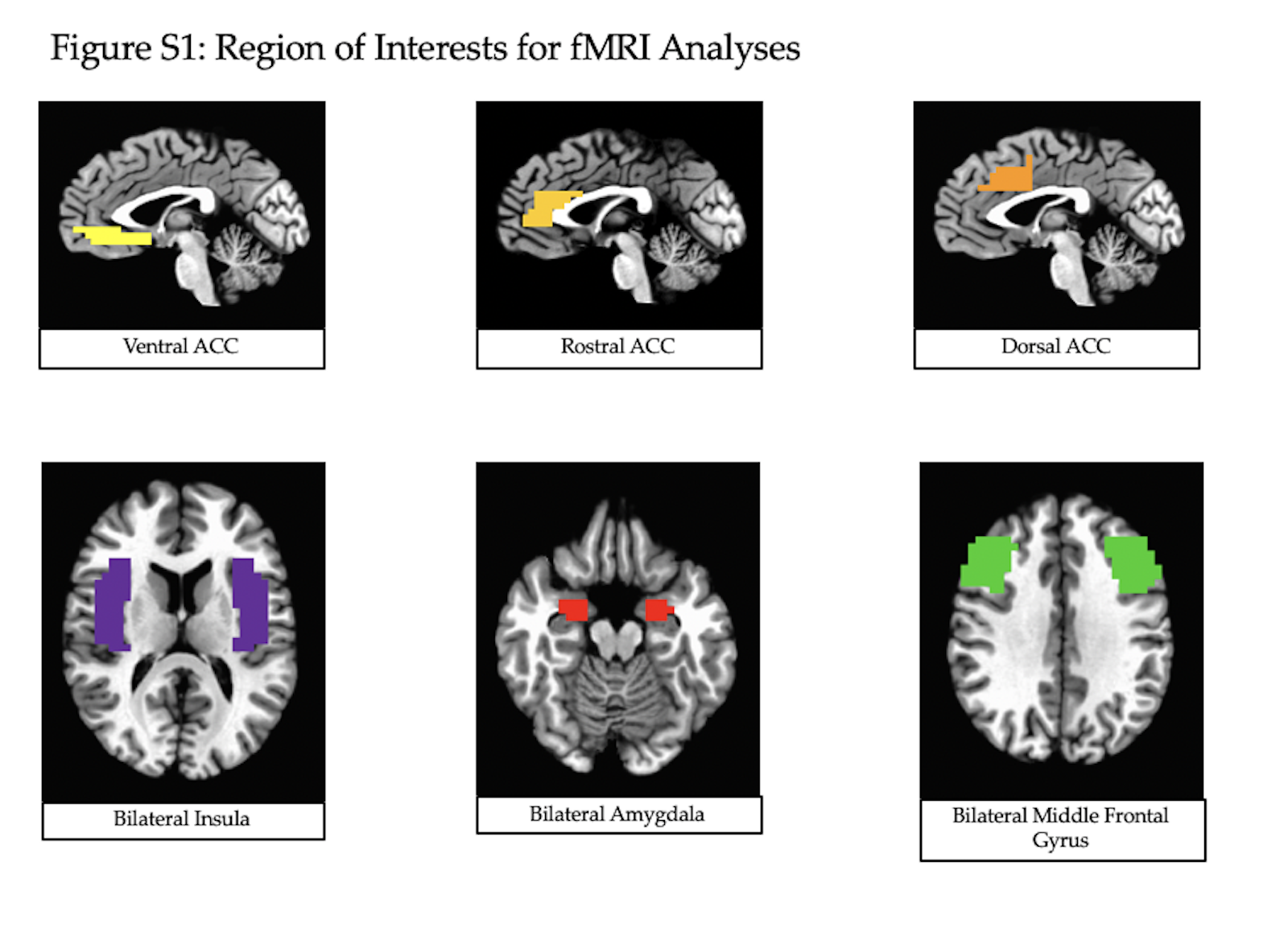

Supplement: Supplementary file 2 [file Image_1.TIFF]
